# Supplementary material for: Maturity Assessment of Different Table Grape Cultivars Grown at Six Different Altitudes in Lebanon
Source: Plants (Basel). 2023 Sep 12;12(18):3237. doi: 10.3390/plants12183237 (PMC10536932; doi:10.3390/plants12183237)
Supplement: Supplementary file 1 [file plants-12-03237-s001.zip › Supplementary Fig. S1_Cultivars.pdf]

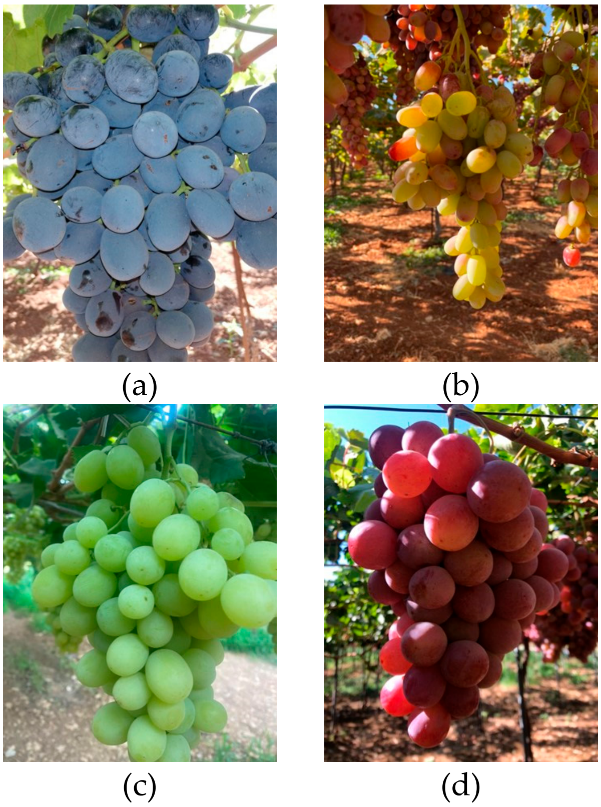

Fig. S1. Photos of table grape cultivars included in this study. (a) Bleak Pearl; (b) Crimson Seedless (immature cluster); (c) Superior Seedless; (d) Red Globe.
