# Supplementary material for: Maturity Assessment of Different Table Grape Cultivars Grown at Six Different Altitudes in Lebanon
Source: Plants (Basel). 2023 Sep 12;12(18):3237. doi: 10.3390/plants12183237 (PMC10536932; doi:10.3390/plants12183237)
Supplement: Supplementary file 1 [file plants-12-03237-s001.zip › Supplementary Table S1_Locations.pdf]

Table S1. Coordinates and altitudes of the geographical locations of vineyards corresponding to each cultivar of table grapes grown in Lebanon.

		Vineyard location					
		El-Qaa (QAA)	Mansourah (MAN)	Zahle (ZAH)	Kfarzabad (KFZ)	Kfarmeshki (KFA)	Baalbeck (BAA)
	Altitudes (m asl)	650	900	950	1000	1100	1150
	Coordinates	33°33'54"N 36°43'45"E	33°70'75" N 35°77'00"E	33°84'16"N 35°94'09" E	33°75'02.3"N 35°96'44" E	33°51'74.1"N 35°75'03" E	33°99'27 "N 36°13'71" E
Cultivars	Black Pearl	x			x	x	x
	Crimson Seedless			x		x	x
	Superior Seedless	x		x	x	x	
	Red Globe	x	x	x		x	x

m asl: meters above sea level.
