# Supplementary material for: Maturity Assessment of Different Table Grape Cultivars Grown at Six Different Altitudes in Lebanon
Source: Plants (Basel). 2023 Sep 12;12(18):3237. doi: 10.3390/plants12183237 (PMC10536932; doi:10.3390/plants12183237)
Supplement: Supplementary file 1 [file plants-12-03237-s001.zip › Supplementary Table S2_Agricultural practices.pdf]

Table S2. The agricultural practices applied in the vineyards under investigation.

**Location of the vineyard**	**Trellis system**	**Thinning**	**Fertilization program (per tree)**	**Soil type**
El-Qaa (QAA)	Y trellis	Gibberellic acid+manual	▪ 12/61/0: 100 g<br>▪ 20/20/20: 200 g<br>▪ Calmag: 300 g<br>▪ K_2SO_4: 200 g<br>▪ 7/6/33: 50 g	Clay-sand
Mansourah (MAN)	Pergola	N/A	▪ 12/12/17: 1kg<br>▪ NH_3: 200 g<br>▪ 20/20/20: 200 g<br>▪ K_2SO_4:150 g<br>▪ SMG:150 g	clay
Zahle (ZAH)	Pergola	Gibberellic acid+manual	▪ 12/12/17: 1kg<br>▪ K_2SO_4: 200 g<br>▪ Calmag: 250 g	clay
Kfarzabad (KFZ)	Pergola	manual	▪ 12/12/17: 0.5 kg<br>▪ K_2SO_4: 200 g<br>▪ 20/20/20: 250 g<br>▪ $MgSO_4$: 200 g<br>▪ Calmag: 200 g	Clay-silt
Kfarmeshki (KFA)	pergola	N/A	▪ 20/20/20:150 g<br>▪ $Ca(NO_3)_2$:150 g<br>▪ SMG:150 g<br>▪ K_2SO_4: 150 g	Silt soil
Baalbeck (BAA)	Pergola	N/A	▪ 12/12/17: 2 kg<br>▪ Urea: 100 g	Clay-sand

N/A: not aplicable.
