# Supplementary material for: Maturity Assessment of Different Table Grape Cultivars Grown at Six Different Altitudes in Lebanon
Source: Plants (Basel). 2023 Sep 12;12(18):3237. doi: 10.3390/plants12183237 (PMC10536932; doi:10.3390/plants12183237)
Supplement: Supplementary file 1 [file plants-12-03237-s001.zip › Supplementary Table S3_Environmental conditions.pdf]

Table S3. The environmental conditions recorded from each location from June to October 2021.

		Air Temperature (°C)			Relative Humidity (%)			Dew Point (°C)		Precipitation (mm)	Wind Speed (m/s)		VPD (kPa)		Air Pressure (kPa)		
**Month**	**Location**	**avg**	**max**	**min**	**avg**	**max**	**min**	**avg**	**min**	**sum**	**avg**	**max**	**avg**	**min**	**avg**	**max**	**min**
**JUN**	QAA	23.1	32.4	12.9	60.1	95.8	22.9	12.8	6.2	0.0	0.7	3.3	-	-	95.1	95.3	94.9
	ZAH	20.9	29.6	10.9	56.0	93.7	22.2	9.7	2.1	0.1	-	-	-	-	-	-	-
	KFZ	21.7	30.1	12.3	48.5	79.0	19.9	8.3	1.5	0.0	1.5	3.9	1.7	0.4	90.5	91.1	84.4
	KFA	21.1	29.4	13.1	50.2	79.0	23.8	8.9	1.4	0.0	-	-	1.5	0.4	-	-	-
	BAA	22.0	32.6	10.6	41.1	76.5	13.1	5.5	-2.3	0.0	-	-	1.9	0.3	-	-	-
**JUL**	QAA	26.9	36.6	17.0	59.9	96.1	21.7	16.0	8.1	0.0	0.8	3.5	-	-	94.7	94.9	94.5
	ZAH	24.3	33.3	13.9	54.0	94.6	22.9	12.4	6.2	0.0	-	-	-	-	-	-	-
	KFZ	25.4	34.5	15.5	44.1	70.7	20.0	10.7	5.3	0.0	1.5	4.5	2.1	0.5	90.7	90.8	90.5
	KFA	25.0	33.1	17.6	47.2	72.8	24.5	11.6	3.9	0.0	-	-	1.9	0.6	-	-	-
	BAA	26.0	37.0	14.9	37.4	66.6	13.3	8.1	1.5	0.0	-	-	2.5	0.6	-	-	-
**AUG**	QAA	27.1	36.8	17.1	57.0	93.8	19.7	15.3	7.5	0.0	0.7	3.5	-	-	94.8	95.0	94.7
	ZAH	24.2	34.0	13.5	56.2	96.7	20.3	12.4	4.6	0.0	-	-	-	-	-	-	-
	KFZ	25.4	35.2	15.1	43.9	72.1	17.2	10.2	3.7	0.0	1.4	4.3	2.2	0.5	90.8	91.0	90.7
	KFA	24.8	33.3	17.4	48.7	76.8	23.7	11.9	5.1	0.0	-	-	1.8	0.5	-	-	-
	BAA	25.7	36.7	14.6	37.2	66.7	11.9	7.6	0.1	0.0	-	-	2.5	0.6	-	-	-
**SEP**	QAA	23.0	32.2	14.5	61.3	91.2	26.3	13.6	8.1	0.1	0.6	3.0	-	-	95.1	95.3	95.0
	ZAH	20.2	29.8	10.6	64.1	98.2	24.3	11.2	5.0	0.0	-	-	-	-	-	-	-
	KFZ	21.0	30.4	11.5	53.1	81.9	21.1	9.3	2.9	0.0	1.5	4.3	1.4	0.3	91.1	91.2	90.9
	KFA	20.7	29.5	13.7	58.0	85.0	27.4	10.9	5.3	0.1	-	-	1.2	0.3	-	-	-
	BAA	21.1	31.3	11.2	47.5	77.2	16.7	7.3	0.0	0.0	-	-	1.6	0.3	-	-	-
**OCT**	QAA	18.4	27.6	10.6	59.4	87.6	26.7	8.9	4.5	0.0	0.3	2.5	-	-	95.6	95.8	95.4
	ZAH	17.0	26.7	8.1	62.2	97.2	25.4	7.8	3.1	0.1	-	-	-	-	-	-	-
	KFZ	17.5	26.7	8.9	52.3	79.2	23.7	6.1	1.8	0.1	1.2	3.6	1.2	0.3	91.4	91.5	91.2
	KFA	18.0	26.6	11.7	53.0	80.8	25.1	7.1	1.8	0.2	-	-	1.1	0.3	-	-	-
	BAA	16.5	26.7	7.7	51.6	79.6	21.0	4.8	-0.3	0.2	-	-	1.1	0.2	-	-	-

QAA (El-QAA, 650 m asl); ZAH (Zahle, 950 m asl); KFZ (Kfarzabad, 1000 m asl); KFA (Kfarmeshki, 1100 m asl); BAA (Baalbeck, 1150 m asl).
